# Dual signaling cascade regulating gut-lung axis in Interleukin-6/Interleukin-17 for NSCLC immuno pathogenesis

**DOI:** 10.3389/fimmu.2025.1649517

**Published:** 2025-11-25

**Authors:** Riya Khilwani, Shailza Singh

**Affiliations:** Systems Medicine Laboratory, Biotechnology Research and Innovation Council-National Centre for Cell Science, NCCS Complex, Savitribai Phule Pune University, Pune, India

**Keywords:** IL-6, IL-17, immunopathogenesis, gut lung axis, CRISPR

## Abstract

Non-small cell lung cancer is the leading cause of death globally, affecting both men and women. Emerging evidence has highlighted the apparent role of gut microbiota in reshaping the lung microbial community. Notably, imbalances in the gut microbiome disrupt lung physiology, which increases an individual’s susceptibility to lung diseases. The homing of gut residents to pulmonary sites prompts tumorigenic processes by altering microbial synergism that metabolically reprograms immune effectors to complement tumor growth. Nevertheless, the additive effect of microbiomes induces immune-responsive mechanisms that excessively induce IL-6 and IL-17 at the inflamed site. Consequently, perturbations in cytokine pool boost inflammatory responses toward a pro-tumor effect, implying cytokine duality and the role of these interleukins in regulating gut-lung crosstalk. Inflammation is a natural host defense mechanism activated against foreign stimulants to mount an immune response. At later stages, the inductive effect of IL-6/17 triggers inflammasome assembly where their accelerated response induces lung epithelial damage, leading to cellular transformation. This implies that the unexplored interconnections between microbiomes and interleukin biology influence immune dynamics that regulate the processes of neoplastic transformation. Here, in this comprehensive review, we comment on the gut-lung crosstalk along with the role of resident microbes in generating immunological responses. Besides, we discuss the IL-6/17-mediated activation of the inflammasome in attuning tumoral immunity. These dictate the potential of microbiotal lifeforms in generating inflammatory responses, which can therefore serve as potential diagnostic markers in NSCLC.

## 1 Introduction

Recent investigations into microbiota have unveiled the intricate tapestry of beneficial microorganisms residing within the human body. These commensal bacteria play a physiological role in regulating cellular homeostasis by interacting with microbial communities in various bodily habitats, including the oral cavity, gastrointestinal tract, respiratory system, urogenital tract, skin, and the host’s immune system (1). The mucosal surfaces, teeming with a diverse microbial ecosystem, including bacteria, viruses, fungi, archaea, and phages, maintain a delicate balance responsible for orchestrating immune activation and overall health (2). Previously, the interaction between the gut and the oropharyngeal microbiomes has been the subject of intense research, while the lungs were considered germ-free. Hence, the lung microbiome remains overlooked by the scientific community. However, with the advent of advanced sequencing technologies like next-generation sequencing (NGS), researchers have shifted their focus to the lung microbiome, realizing its complex ecosystem comprising of bacteria, viruses, and fungi, after which the relationship between the gut and the lung microbiomes has been studied extensively in human context (3). Precisely, the gut-lung axis is a multi-layered bidirectional communication governed intricately at immunological levels between two organ systems, dynamically influencing the metabolic and pathological facets of gut and lung development. Lung homeostasis and immune regulation are determined primarily through microbial ecology and related metabolites, defining host’s immune-responsive states. While the composition of the lung microbiome differs greatly between diseased and healthy individuals, its role in lung health and development is evident. The exact mechanism underlying this influence still remains unknown and requires a significant understanding of disease-microbiome interactions and microbial signatures to potentiate microbiome-based therapy (4).

The gut microbiome, which is a varied collection of bacteria inhabiting the GI tract, plays a key role in sustaining pulmonary health while regulating the host’s physiological perturbations. The stable interconnection between the gut microbiota and the resident immune cells reverses with gut dysbiosis, compromising the lung profile and leading to immune imbalance. Dysbiotic events in the gut have been identified as a hallmark of cancer, wherein a few of many microbial populations skew the immune profile to harbor a suppressive milieu at the wounded site (5). An alternative cause that works additively in enhancing lung cancer susceptibility is microbiome homing to lungs and other sites, potentially leading to homeostatic imbalance and adverse health consequences (6). Lung cancer, which is proven to be lethal cancer, elevates mortality risk through releasing cancer-promoting metabolites conferring immune resistance. While how the gut-lung axis rewards malignant transformation during later stages needs to be researched. As per our current understanding of the existing interconnections between microbiome and lung health, it relies on the inherent abilities of the gut microbiota in altering drug effectivity and immunotherapies. As an effect of antibiotic intake, the expansion of varied bacterial populations has been shown to diminish the effect of immune checkpoint inhibitors in NSCLC patients, altogether resulting in reduced clinical efficacy and shorter overall survival rates (7). In line with this fact, the translocation of pathogenic organisms into lungs favors Th2 immune responses over Th17 cell-mediated immunity, deregulating Th17/T-reg immune axis and pathological events. In a study, mice infected with *Klebsiella* species reduced innate immune populations of macrophages and neutrophils while fostering an immunosuppressive T-reg phenotype in lungs. Additionally, *Lactobacillus* probiotic treatment restricted lymphocyte expansion, contributing to hyperactive lesions in lungs. Combinely, these compromises lung security since the stability of the tolerant phenotype sustains the immunomodulatory effect and hence, the intestinal inflammation (8, 9). At cellular levels, the transduction of pathogenic signals to immune effectors is commenced upon cytokine duality, where the transactivation of IL-6 and IL-17 cytokine combinations exacerbates pathogenic responses and subsequently systemic inflammation. This is assisted through the differentiation of immune subsets like effector T-cells, pathogenic Th17 cells, and T-regs, which triggers inflammatory responses at the expense of immunologic effect. In patients with IBD, macrophage activation elicits the release of pro-inflammatory cytokines, including IL-1β, TNF-α, IL-18, and IL-6, contributing to Th17 differentiation and pathological responses. As an effect, an abrupt release of pro-inflammatory cytokines alters microbiome composition that is responsible to shape cytokine dynamics (10, 11) and the pleiotropic behavior of IL-6/17 towards organ dysfunction.

**Figure 1:**
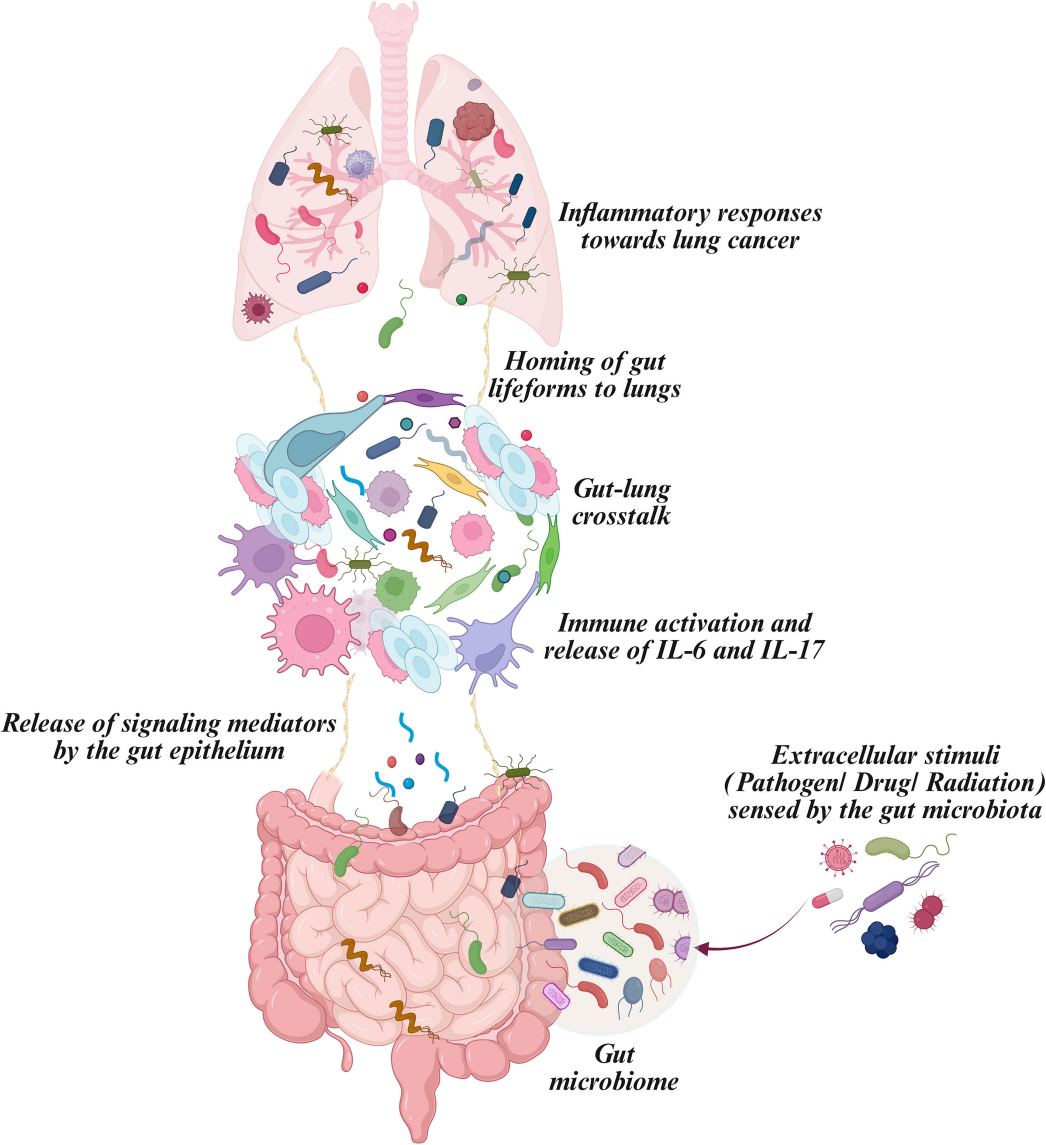
The gut-lung axis and microbiota-driven cytokine activation. Gut commensals’ response to extracellular stimuli releases microbial metabolite, which home to lung sites. The homing of microbial materials in lungs induces immune-cytokine activity, which synthesizes IL-6 and IL-17, resulting in pulmonary inflammatory responses.

In the context of lung cancer, the immune sentinels, particularly macrophages and T cells, regulate disease severity. Under hypoxic conditions, T-lymphocytes enter the tumor-permissive environment that recognizes foreign entities as self-antigens, generating a T-reg pool and the pro-tumor effect (12). The immune activation signals transduce T-cell responses that trigger the synthesis of pathogenic Th17-derived IL-6 and IL-17. Their additive effects in the tumor microenvironment upregulate inflammasome mediators that assemble into a complex and transduce the phenotype of alternative macrophages. As a consequence, perturbations in macrophage biochemistry contribute to excessive inflammation and tissue damage. Recalling the cause that facilitated alternative activation of macrophage populations is the alterations in the composition of the gut microbiome. Macrophages, being primary defenders, exhibit phenotypic plasticity in response to environmental cues, so-called polarization. Under-stressed conditions, IL-4 and IL-13 induce macrophages to secrete anti-inflammatory molecules like arginase and IL-10 that have an important role in tissue remodeling and neovascularization (13). Given that changes in the gut microbiome impact the lung microbiota and influence drug effectiveness, combining microbial therapy as an adjunct to chemotherapy could potentially enhance treatment outcomes. The strategic way to combat this rising inflammation could be gut modulation, wherein physiologically altering microbiota enhances immunotherapeutic responses by regulating inflammatory effects. Current interventions for gut modulation include microbial engineering, prebiotics, probiotics, and dietary modifications (14). In this comprehensive article, we lay down insights on the gut-lung axis, a detailed perspective on the existing correlation between gut microbiome and lung cancer, with a special focus on the role of pleiotropic cytokines, IL-6 and IL-17, in regulating this axis in NSCLC development (**Figure 1**).

## 2 Dysbiosis bridges the gut and lung to fuel NSCLC metastasis

Non-small cell lung cancer, a leading cause of lung cancer deaths, is significantly influenced by the microbial communities inhabiting the gut and lungs. These microbes play critical roles in regulating metabolic, immunological, or inflammatory processes during lung cancer development, making them important diagnostic markers. Typically, an imbalance in bacterial microflora escalates immunosuppressive mechanisms and influences the metastatic spread of lung cancer cells. Upon analyzing the microbial characteristics in LUAD and LUSC patients, the SHOGUN RNA-seq dataset identified bacterial sequences that predominantly belong to the phyla *Actinobacteria*, *Chlamydiae*, *Candidatus*, *Bacteroidetes*, *Firmicutes*, and *Proteobacteria*, signifying their role in lung tumors. Although these bacteria are common to primary tumor sites and solid tissue samples, bacterial counts are significantly found to be higher between primary sites. Volcano plots highlighted differential bacterial genera between these sites, wherein genera such as *Alcanivorax*, *Cyanothece*, and *Sulfolobus* were specifically enriched in LUAD (15). A separate study indicated substantial differences in the microbial composition at the phylum level between tumor and adjacent non-tumor sites. Notably, the tumor tissues exhibited a higher abundance of *Haemophilus* and *Streptococcus* compared to adjacent groups. Additionally, *Lactobacillus*, *Neisseria*, *Streptococcus*, and *Pasteurella* were found to be significantly enriched in the tumor group, implying their roles in cancer progression (16). On the contrary, the role of *Lactobacillus* has been shown to have probiotic properties that mitigate cancer-promoting effects in normal tissues. This highlights its role in both promoting and rescuing cancer-specific traits during later and early stages, respectively (17). Additionally, the gut’s fungal richness intensifies the processes of neoplastic transformation. Higher frequencies of *Candida albicans*, *Aspergillus niger*, and *Aspergillus fumigatus* have been shown to be associated with increasing clinical severity. Microscopic examination of lung tissue specimens revealed the evidence of fungal colonization and septate hyphae in few lung cancer patients (18). In a study by Kim et al., the consumption of antibiotics induces the overgrowth of gut fungus that promotes airway inflammation by elevating plasma prostaglandin E_2_ (19, 20). We could therefore comment that microbial dysregulation regulates the tumor microenvironment and behavioral ecology of immune subsets residing within tumors. Consequently, the release of inflammatory mediators transforms lung developmental programs and compromises respiratory health. However, how the intricate relationship between the triad of microbial communities, immune activation, and cytokine production contributes to lung pathophysiology is discussed below.

### 2.1 Gut microflora versus lung microflora

The human GI tract is home to a diverse community of bacteria called intestinal flora, which anatomically functions to regulate gut immune functioning and has a significant impact on the gut ecosystem. However, a change in the composition of gut microbiota has been implicated in lung cancer. A recent study reported comparatively higher levels of *Fusobacterium, Bacteroides, Bacillus, Enterococcus, Prevotella, Streptococcus, Lactobacillus, and Veillonella* in patients, while *Escherichia coli* and *Enterobacter* were significantly lowered, suggesting them as diagnostic biomarkers in lung cancer (21–23). Alterations in the gut microflora affect prebiotic life forms thereby modulating drug metabolism, immune responses, and checkpoint immunotherapy. Early preclinical research reported the oral administration of *Bifidobacterium* in enhancing anti-tumor immune responses by eliciting DCs for promoting cytotoxic roles of CD8+ T-cells, aiding in ICIs effectivity (24). Consequently, the reversal in immune phenotypes affects the differentiation program leading to immune regulation, potentially influencing lung carcinogenic events. Nevertheless, a retrospective study by Tomita et al. on ~120 NSCLC patients evaluated the therapeutic intake of *Clostridium butyricum* in prolonging the overall survival rate of patients when given in adjunct with immune checkpoint blockers (25). Therefore, we could say that the changes in gut biodiversity define lung health and treatment outcomes, which could be leveraged to diagnose the cellular metastatic spread. In contrast to gut microbiomes, lung microbiota in a healthy adult is dominated by *Firmicutes, Proteobacteria*, and *Bacteroidetes*, all of which define the lung’s pathological condition (26, 27). Their ecological balance in the healthy ecosystem is characterized by their ability to grow, multiply, and reproduce, which compromises upon their low count in diseased conditions. Additionally, investigations through bronchoscopic analysis reveal the elevated levels of gram-positive and negative bacteria, including *Escherichia coli, Haemophilus influenzae, Staphylococcus*, and *Enterobacter*, in lung cancer patients, which suggest the role of these microbes in immunomodulation. Along with these bacterial species, the increasing titer of *Neisseria*, *Veillonella, Capnocytophaga*, and *Selenomonas* correlates with lung adenocarcinoma, implying the specificity of these genera in lung cancer (28). While other microorganisms also play a role in lung cancer pathogenesis, *Enterobacter, Veillonella*, and *Escherichia coli* are among the commonest bacterial communities that host the mucosal surfaces of the gut and lungs. This could be the underlying reason for triggering chronic illness, where similar microbiotal flora stimulates the growth of regulatory subsets, ultimately leading to the elimination of symbiotic microbiomes in the local area. As an effect, the induction of anti-inflammatory cytokines initiates pulmonary transformative events, which transit cells towards tumor initiation. This microbial synergism between two distantly located organs suggests similar embryological origin, which implies the importance of organ reciprocity in regulating immune perturbations during cancer development.

### 2.2 Microbiome-mediated cytokines drive immune modulation in cancer

Symbiotic residents in the gut and lungs are important to the regular maintenance of homeostatic equilibrium. Microbial dysbiosis in these local regions alters the bacterial count, exaggerating immune responses toward inflammatory processes. Results from mediation MR analysis described the causal role of the gut microbiome in altering circulating cytokine levels. The abundance of bacterial populations in the stool of lung cancer patients significantly increased the levels of IL-20, IL-24, CCL-8, and TGF-α in LUAD and LUSC patients, indicating the role of these proteins in bridging the association between gut ecology and lung cancer (29). Since the homing of gut lifeforms affects lung microbial ecology, higher bacterial burden in lung tumors is associated with reduced CD8+ T-cell levels and increased PD-L1 and Ki-67 expression (30). As a result of high biological activity in the immune repertoire, including macrophages, NK cells, and CD4+ T-cells, an imbalance in the cytokine-inflammatory axis contributes to heightened inflammatory response, cytokine storm, and systemic inflammation (31). These changes in immune behavior signify the role of innate and adaptive immune arms in lung transformation, which drives immune sentinels to achieve modulatory states. The innate immune system, which is the body’s first line of defense, activates PRRs and signals downstream when sensed by stimulants such as allergens, toxins, and/or any particulate matter. Usually, microorganisms drive inflammatory responses by activating TLR2 and TLR4, thereby enhancing the adhesion properties and metastatic potential of cancer cells, all of which affects prognostic outcome in NSCLC patients (32). Immune reprogramming thus alters cellular dynamics, which influence inflammatory events and constitutively secrete regulatory proteins. Among all immune populations, epithelial cells and macrophages are primary defenders that colonize lung airways and provide defense against foreign invaders. However, the activation of B and T lymphocytes, majorly ILCs, NKT cells, and γδ-T cells, tones lungs immunity via eliminating cellular insults. DCs, which are the interconnecting link between immune arms, stimulate the expression of CD4 and CD8 co-receptors and maintain tissue homeostasis via activating repair mechanisms. Following this, the activation of regulatory T cells counteracts the excessive release of inflammatory mediators and promotes immune-suppressive mechanisms to orchestrate immune balance. Additionally, the activation of NK cells and monocytes primes cellular transformation, which correlates to the role of alternative immune populations (M2 macrophages) in advocating the tumorigenic effect. As an effect, the naïve T cell differentiation towards the pathogenic CD4+ Th17 lineage reflects the positive feedback mechanism between immune-responsive and modulatory subsets that intensifies inflammatory responses and the process of tumor development. Of all the CD4+ T-cell subtypes, Th17 cells are intensively studied because of their paradoxical roles in protection versus pathology. Naturally, the intestine harbors distinct Th17 populations; however, their roles rely on the bacterial type that stimulates T-cell differentiation. In the intestine, Th17 differentiation elicited by filamentous bacilli displays non-inflammatory properties, while others trigger pathogenic Th17 differentiation dependent on IL-23 receptor activation (33–35).

Gut bacteria influence lung health by releasing substances that travel through the bloodstream, connecting the gut and lungs. The process of neoplastic transformation starts when the immune system compromises, causing the homing of gut microflora to lung sites. Initially, the activation of monocytes, natural killer cells, and dendritic cells tunes immune responsive mechanisms to attain homeostatic states. This is assisted through the release of pro-inflammatory cytokines, IFN-γ and IL-12, which shape the immune landscape and subsequent immune responses in restoring the inflammatory pool. As an effect, activating signal transduces myeloid effectors such as monocytes to generate inflammatory initiators, IL-1β and IL-23. This marks the induction of the T-cell-mediated responses, which produce cytokine combinations, IL-6 and IL-17, to combat the rising inflammation. However, as the tumor progresses, the excessive release of these cytokines sustains Th17 differentiation in the redox environment. Studies indicate that RORγt gene deficiency in young NEMO^IEC-KO^ mice does not improve colitis development, signifying Th17 responses to be effective during later stages of disease development. Nonetheless, IL-23-dependent activation of IL-17A in older NEMO^IEC-KO^ mice indicates that Th17 cells might drive chronic inflammatory processes (36, 37). Research evidence highlights IL-6 and IL-17 as diagnostic markers in lung cancer; of which IL-6, more than IL-17, was found to be significantly higher in the serum of patients with adenocarcinoma. Consistently, chemotherapeutic drugs did not reduce IL-6 levels as compared to IL-17 levels in response to treatment, suggesting the diagnostic roles of these proteins in NSCLC (38). During later stages, these cytokines behave pleiotropically, which reverses the immune profile towards generating a pro-tumor effect. Nevertheless, in the TME, the release of angiogenic factors, chemokines, and suppressive proteins by tumor cells induces differentiation of M2 macrophages, cancer-associated fibroblasts, MDSCs, and endothelial cells, all of which drive immune-tolerant mechanisms. The activation of STAT3 tyrosine signaling promotes M2 ploarization of tumor-associated macrophages, while indirectly influencing EMT processes in NSCLC cells (39). Altogether, this shift in the immune environment, which is driven by the interplay between immune cells and inflammatory mediators, therefore contributes to the processes of tumor initiation and development.

**Figure 2:**
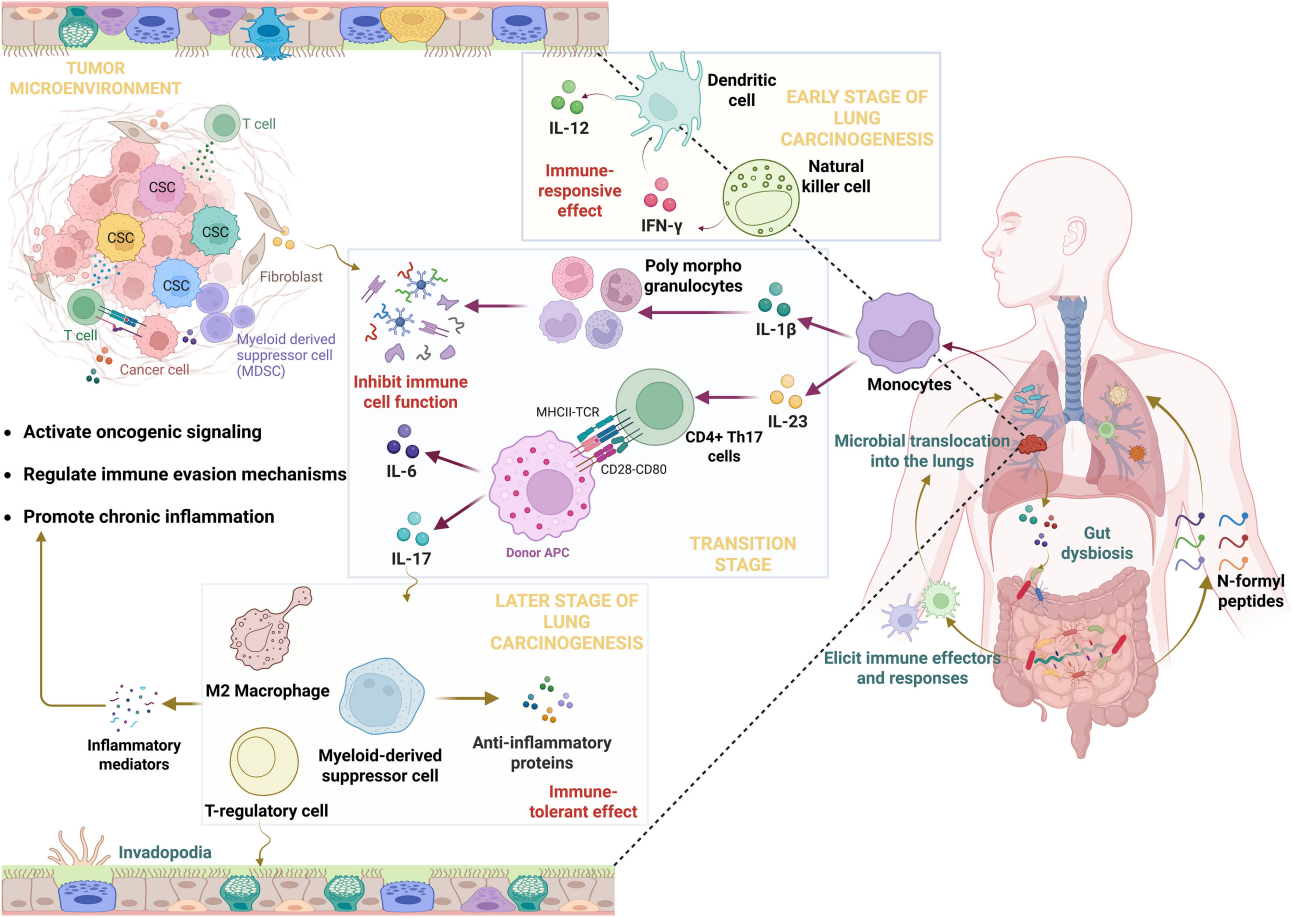
The intricate dance: The gut-lung-microbiome-cytokine axis in lung cancer metastasis. The figure illustrates a bidirectional communication between the gut and the lungs, where signals originating from the gut influence the behavior of lung cells. An imbalance in the gut’s microbial composition leads to the release of bacterial products that trigger immune responses within the lungs. In the initial phases, the activation of monocytes, natural killer cells, and dendritic cells leads to immune-responsive mechanisms. However, the sustained release of cytokines promotes the production of other inflammatory mediators while also fostering immunosuppressive cell types. In later stages, the differentiation of immune-modulating cell populations and the amplified effect of signaling molecules activate oncogenic pathways, ultimately determining the fate of lung cells. Nonetheless, signaling molecules from the cancer cells themselves contribute to establishing chronic states within the lungs.

While the role of lung microbiota in cancer development is not increasingly recognized, the existence of one theory explains the direct involvement of lung bacteria in immune disruption. The primary mechanism by which lung microbiota contributes to cancer development is not only restricted to specific conditions but is influenced by various factors, such as environmental cues and underlying chronic conditions. This chronic illness triggers microbial dysbiosis that increases bacterial colonization and also sustains tumor physiological processes. Majorly, how the host immune system responds to the bacterial community is through their byproducts that recruit immune effectors to the site of lung inflammation. Among the list of soluble components that are secreted in the extracellular milieu, the release of N-formyl peptides serves as a potent chemoattractant that stimulates monocytes and polymorphonuclear leukocytes to further the inflammatory process. Evidence states that formyl-peptide receptors are involved in myeloid cell trafficking during infection and inflammatory responses in cancer. However, Fpr2, one of the few Fpr receptors, is shown to be involved in healing wounds at anatomic sites that prevent damage to healthy nearby cells (40, 41). As an effect, the physiological changes in the alveolar cells negatively impact the cells lining the lung epithelium. This triggers the release of inflammatory molecules like IL-6 and IL-8 (a cytokine responsible for neutrophil chemotaxis), which transduces signaling mechanisms for tumor development (42). Primarily, the activation of NFkB and associated transcription events induces IL-1β and TNF-α to prompt the proliferative, invasive, and migratory events (43). Instead, the activation of other cytokines and interleukins contributes to tumorigenesis. STAT-3, which is a transcription factor activated through cytokine signaling, induces the differentiation of respective T-helper lineages depending on the context; its polarization towards tumor regulation attenuates DNA repair processes, leading to genomic instability (44). Despite this, gram-negative bacterial byproducts have been linked to apoptosis resistance and metabolic alterations. The change in the metabolic profile of lung cancer patients, such as the increase in amino acid and lipid metabolism, impacts gene expression patterns in respiratory cells (45). These lead to the activation of anti-apoptotic factors, cell cycle proteins, and survival proteins that regulate pathological processes in cancer development. This tells us that the microbial profile and their related byproducts have the potential to alter the immune balance that perturbs the tumor microenvironment and favors immune-suppressive mechanisms in advancing tumor state (**Figure 2**).

### 2.3 Dual function cytokines: IL-6/17 pleiotropy and transformative effects

As discussed, dysbiosis in the gut and lungs induces inflammatory responses that activate the IL-6/17 signaling axis to favor the host’s suppressive events, indicating the indirect and pleiotropic role of IL-6 and IL-17 in linking microbiomes to inflammation. At initial stages, IL-6 and IL-17 are inflammatory mediators that are secreted to promote wound healing effects and eliminate foreign antigens. The dominance of pro-inflammatory characteristics of these cytokines over regulatory cytokines contributes to immune balance and homeostasis. During the time when microbial level changes and/or the resident microbes are replaced with foreign invaders, host responses equilibrate the immune surroundings. IL-6 and IL-17, along with other pro-inflammatory mediators, rescue these immune perturbing effects. However, when the host immune system weakens, the constitutive release of IL-6 and IL-17 contributes to cellular changes and transformation. In mice, microbiome-mediated cytokine release induces FOXP3+ T-reg differentiation that exerts an immunosuppressive effect during lung metastasis. This enriches lower airways with oral commensals that elicit IL-6 and IL-17 signaling mechanisms and hyperactivate lung inflammatory events (46). Research demonstrated that following intravenous injection of the mouse LLC1 cells into the tail vein, lung metastases were substantially less common in an IL-17^-/-^ mice model of lung adenocarcinoma than in the wild-type mice (47). Additionally, in the LUAD mice model, IL-17A was demonstrated to promote tumor growth by inducing IL-6. Their synergistic effect activates NFkB and STAT3 activation, promoting cellular growth and survival. In the context of microbiome-mediated IL-17 activation, the release of CCL20 by the filamentous bacilli in the intestine draws IL-17 to the lung sites, which impairs Th17 homeostasis, causing lung disease (48–50). Therefore, it’s the microbial effect that triggers immune reactions and perturbs the cytokine pool towards releasing IL-6/17 cytokines.

Being pleiotropic, IL-6 displays both pro-inflammatory and anti-inflammatory behavior during cancer development. At initial stages of cellular transformation, it is released by monocytes and macrophages in response to cytokines secreted by tumor cells. The canonical IL-6 signaling starts with the binding of IL-6 to its receptor present on the membranes of white blood cells, epithelial cells, and also liver cells that expresses pro-inflammatory cytokines for antigen clearance. IL-6 binding with soluble forms of receptors (sIL-6R) triggers lymphocyte differentiation and neutrophils chemotaxis to the wounded site. IL-6, which helps regulate inflammation, plays a significant role in the development and progression of non-small cell lung cancer. The cytokine storm seen in NSCLC patients is triggered by the JAK kinase family, which activates various signaling pathways, including MAPK and PI3K-AKT. These pathways lead to the release of cytokines that promote tumor growth, cell division, invasion, metastasis, and angiogenesis, allowing the tumor to evade the host’s immune response. Therefore, the coordinated inhibition of PI3K and MAPK signaling is necessary to induce apoptosis, reduce cellular proliferation, and promote anti-tumor activity (51). Majorly in lung, colon, breast, and ovarian cancers, it acts as an autocrine factor that favors anti-apoptotic responses and stimulates cellular proliferation and survival. Interestingly, patients with lung cancer have been shown to have the highest IL-6 levels, which positively correlate to its role in cancer progression and therapeutic resistance during tumor development (52). As shown in the study by Qu et al., IL-6-deficient mice induced with K-Ras oncogene initially suppress tumor initiation and regulate lung homeostasis through inducing STAT3 mechanisms, stimulating the effector cytotoxic CD8+ T-cell populations in the redox cytokine milieu. This defines the tumor-suppressing effect of IL-6 during early stages of tumor development. On the other hand, the same study emphasizes how IL-6 promotes tumor growth by favorably regulating it through expressing the cell cycle regulator, CyclinD1, suggesting the pleiotropic roles of IL-6 in lung cancer progression and development (53). To note, IL-6 doesn’t undergo any mechanism of alternative splicing, meaning that the cell-specific post-translational modifications are necessary to achieve IL-6 function. However, in many cancers, the aberrant form of glycosylated IL-6 reduces STAT3 activation, leading to increased activity of the SRC-YAP-SOX2 signaling axis. This alteration in the downstream signaling pathway introduces epithelial-to-mesenchymal transition and metastatic events *in vivo*, sufficing earlier findings on the role of canonical STAT3 in tumor suppression (54). These subsequent processes alter the tumor microenvironment by enhancing the cellular habitats with stromal cells, tumor-associated macrophages, and extracellular matrix. Of all the immune cells present in the TME, TAMs are perhaps the most prevalent phenotypes that modulate tumor surroundings and sustain regulatory states in the pulmonary setting. The induction of immune-suppressive cytokines like IL-4, IL-10, and IL-13 secreted through tumor cells, endothelial cells, and MDSCs transforms classical macrophages towards an alternative phenotype. This is assisted through the anti-inflammatory effects of IL-6 and IL-17 that have been evident in inducing invasive events in lung cancer cells, creating a hypoxic TME and therefore the immune resistance (55). As a consequence, the polarization of monocytes towards the suppressive myeloid lineage triggers transformative events that, being stressed, positively signal the M2 phenotype to induce the macroautophagic response. Autophagy is a conserved physiological defense mechanism that is activated under stressed conditions and sustains nutrient-deprived cells. It is thought to play an important role in polarizing macrophages, wherein the increased autophagy flux triggers the differentiation of alternative macrophage populations to release anti-inflammatory molecules (56). During malignant states, the constitutive activation of IL-6/17 signaling influences the assembly of the inflammasome complex that triggers inflammatory responses and affects the biology of inflamed cancer cells. Therefore, we could state IL-6 and its pleiotropic role positively drives the physiology of growing tumor cells towards achieving chronic states in NSCLC.

Similar to IL-6, IL-17 exerts immune-suppressive effects during later stages of tumor development. In response to IL-23 (a kind of pro-inflammatory cytokine), IL-17 is released by pathogenic CD4+ Th17 cells that activate multiple signaling mechanisms by binding to complex receptors, which may have differing roles in cancer progression. Over the T-cell surface, the formed IL-23/IL-23R complex facilitates the canonical JAK-STAT signaling pathway that induces pathogenic responses in pulmonary lymphocytes. The downstream inductive signals upregulates the transcription factor, STAT-4, that favor the synthesis of IFN-γ in hypoxic milieu. Being an anti-tumor cytokine, the release of IFN-γ provides hosts the immune strength, where its ability helps in counteracting the immune regulatory effects generated by suppressive cytokines. Additionally, the IL-23-mediated activation of STAT-3 induces RORγt, a transcription factor, which is unique to inducing inflammatory responses. Being a crucial cytokine, the expression level of IL-17 at different stages determines the fate of tumor cells. Initially, its role as a pro-inflammatory cytokine serves to eliminate tumor antigens; however, as the cancer progresses, the polarization of naïve CD4+ T-helper cells towards pathogenic Th17 lineage releases IL-17 and also IL-6 cytokines that are important to advance tumor stage and development. In immune-compromised conditions, the excessive levels of IL-17 tune pro-tumoral immunity by stabilizing the phenotypes of suppressive T-regulatory cells, Th2 cells, and also myeloid lineages (MDSCs). These regulatory subsets in the tumor microenvironment are stabilized through upregulation of transcription factors like FOXP3, GATA-3, and STAT-6, all of which translate pro-tumor cytokines to achieve immune-tolerant states. Immune modulation occurs when these transcription factors, along with their induced cytokines, modulate Th1 phenotypes to accomplish immune reversal leading to tolerant states. While how these combinely alters the tumor microenvironment and influences malignant phenotypes requires deeper understanding of the pathogenic role of IL-17 in cancer progression. IL-17 has its receptors on different immune and non-immune cells, including dendritic cells, endothelial cells, epithelial cells, and macrophages. The translocation of IL-17 in M2 macrophages initiates a series of signal transduction events. The induction of MAPK is the key signaling mechanism that is at first activated, followed by the induction of PI3K-AKT and JAK-STAT pathways crucial to tumor development. Crosstalk between these signaling pathways induces AP1, CEBP-β/δ, and Erk1/2, the additive effect of which drives the expression of anti-apoptotic genes together with the proliferation, survival, and migratory proteins, stabilizing the homeostatic equilibrium of M2 macrophages. As an effect, the expression of Bcl-xL, cyclin-D1, and VEGF favors the angiogenic process and imparts survival abilities to the inner growing tumor mass. The synergistic effect of IL-6 and IL-17 has been shown to induce the expression of anti-apoptotic proteins in cancer cells, even at molecular concentrations where the individual cytokine effect is negligible (57). At the clinical settings, the combined elevation of IL-6/17 serves as a stronger prognostic marker for poor survival and progression free survival in patients with lung cancer (58). Despite the direct effect of IL-17 in sustaining tumor growth, the indirect IL-17-mediated activation signals additionally prompt the tumorigenic process. This is assisted through the activation of the master regulator, NFkB, which mediates inflammatory responses by assembling an inflammasome, thereby achieving chronic states. Altogether, these events mark the transformative events where the secreted cytokines and growth factors additively trigger malignant phenotypes and their sustenance in the hypoxic tumor microenvironment. From the above evidence, we could therefore comment that both IL-6 and IL-17, being pro-inflammatory, exert pro-tumor effects during later stages of tumor development, which therefore marks that these cytokines, being different, work synergistically to promote progressive events in lung cancer (59, 60).

## 3 Inflammasome activation in lung cancer: a role for gut-lung communication

Given the link between the gut dysbiosis and inflammation, the interplay between the gut microbiota and inflammasomes is crucial for disease outcomes (61). When pathogenic microorganisms encounter the gut epithelium, it triggers signaling events, leading to the activation of the inflammasome. The interaction between pathogenic microorganisms and the gut epithelium sets off a chain of events that activate inflammasomes as a part of natural defense. Inflammasomes are cytosolic multimeric protein complexes activated through PRRs when sensed by any external stimuli such as pathogens, toxins, and/or any danger signals, and play crucial roles in cell death processes. The exposure of gut epithelium to pathogens triggers the expression of inflammasome proteins, where the extent of inflammation decides cellular phenotypes, contributing to gut homeostasis. Recent research highlights the reciprocal relationship between inflammasomes and the microbiome, where inflammasome activity can influence the composition of the gut microbiota, and conversely, the microbiome can modulate inflammasomes. During the translocation of gut pathobionts to lungs, the alteration in the size of commensal organisms triggers innate immune responses and inflammasome activation. While optimal inflammasome activity is crucial for effective immune defense, excessive or dysregulated inflammasome activation can contribute to various pathologies, including autoimmune diseases, cancer, and other inflammatory conditions. In colitis, oral dysbiosis resulting from *Enterobacter* species induces robust release of pro-inflammatory cytokines as a result of macrophage NLRP3 activation. Additionally, NLRP3-dependent release of IL-1β drives the polarization of immunosuppressive T-cells and guides them to release IL-22, a cytokine involved in invasive growth in many malignancies. Excessive NLRP3 has been shown to alter the levels of oxidizing species, which negatively influence microbiome composition. Thus, a mutual relationship between the microbe and the NLRP3 inflammasome guarantees intestinal homeostasis (62, 63).

For the first time in 2002, the term “inflammasome” was coined to describe a crucial multi-protein complex pivotal for orchestrating innate immune responses against pathogenic stimuli. These complexes, consisting of a sensor protein (NLR), an adaptor protein (ASC), and the effector caspase-1, are expressed across diverse cell types, including immune cells (like dendritic cells and macrophages) and non-immune cells (like epithelial cells, fibroblasts, and intestinal cells). Upon pathogen recognition, inflammasomes activate caspase-1, leading to the maturation of pro-inflammatory cytokines, IL-1β and IL-18, and inducing pyroptosis, a form of programmed cell death. Among all the inflammasomes, the NLRP3 inflammasome has emerged as a key player in regulating inflammatory responses, particularly within the cancer milieu. As a central sensor, NLRP3 initiates signaling cascades by interacting with adaptor proteins, underscoring its critical role in innate immunity and cellular homeostasis. The NLRP3 activation is a two-step process: priming and activation. Initially, signals from DAMPs or PAMPs induce the expression of NLRP3, its adaptor protein ASC, and precursor forms of inflammatory mediators. Subsequent exposure to stimuli such as ATP and bacterial nigericin triggers the activation step, leading to NLRP3 oligomerization and the formation of the inflammasome complex, ultimately culminating in the maturation of inflammatory mediators. Most commonly, stimuli such as NLRP3 relocalization to mitochondria, cathepsin release, and potassium efflux contribute to ASC nucleation (64). Aforementioned is that the constitutive expression of these proteins can modulate the gut microbiota and can lead to the pathophysiology of cancers; the reverse is also true to mediate tumor progression. In colitis, mice infected with *Helicobacter pylori* activate NLRP3 and IL-18 processing, which is sought to protect against colon inflammation (65, 66). Additionally, this could be justified through the study by Seo et al., where the gut pathogen *Proteus mirabilis* releases hemolysin and potentially upregulates NLRP3 by alternatively polarizing macrophages, which signals colonizing bacteria to generate inflammatory responses, exacerbating colitis and associated immune events (67). This highlights the critical role of immune cells, particularly IELs and lamina propria cells within the intestinal mucosa that play a critical role in maintaining epithelial integrity and facilitating biomolecule transport. However, the imbalance can lead to inflammatory damage and cellular dysfunction. While these cytokines are essential for antimicrobial defense, their dysregulated production can significantly contribute to pulmonary pathology. These findings underscore the pivotal role of NLRP3 in orchestrating intestinal immune responses, emphasizing the intricate and interconnected nature of mucosal immune responses and their profound systemic implications.

**Figure 3:**
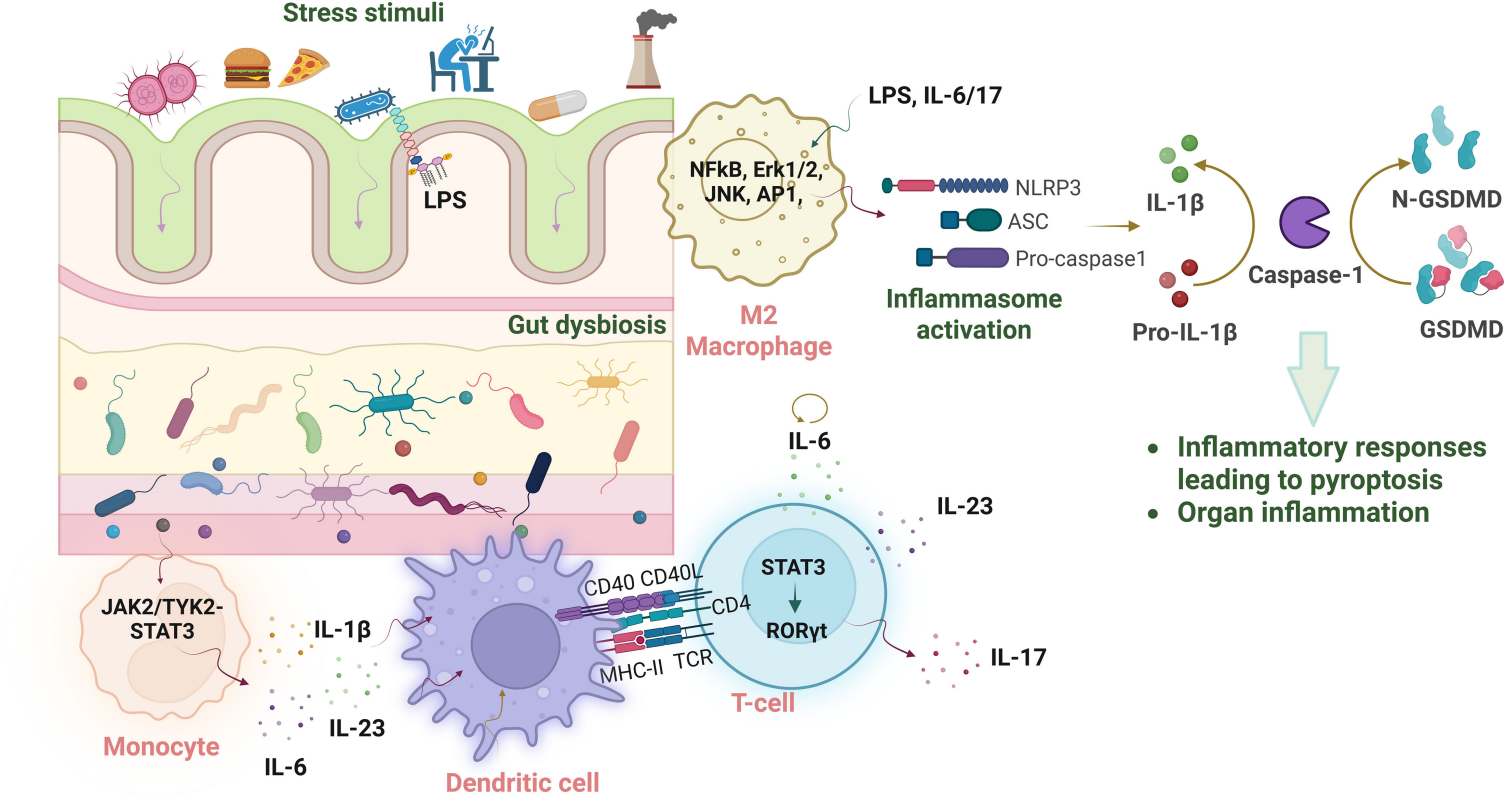
Gut-mediated molecular mechanisms assemble inflammasomes to establish chronic states. Different stress stimuli trigger imbalances in microbial cell populations, leading to immune activation. The exaggerated immune response furthers canonical inflammasome assembly, which upregulates proteolytic enzymes to generate matured forms of inflammatory cytokines, causing organ-specific inflammation.

While the influence of gut microbiota on cancer is well-established, emerging evidence suggests a reciprocal relationship whereby NLRP3 inflammasome activity significantly shapes the gut microbiome and contributes to tissue homeostasis. The critical role of activated T-regulatory cells in maintaining tissue homeostasis following gut modulation supports the evidence that the excessive activation of NLRP3 restores equilibrium while stimulating immune suppressive phenotypes during cellular transformation (66). Given the established correlation between IL-6/17 cytokines, gut microbiota, and inflammasome activation in modulating cellular immunity, here, we attempt to highlight downstream molecular events mediated by NLRP3 that contribute to chronic inflammatory states during lung transformation. The synergistic effect of IL-6 and IL-17 orchestrates immune responses by driving macrophage polarization, which in turn primes the activation of inflammasomes during the initial phases. As the host defenses weaken, signaling pathways are triggered that lead to enhanced NLRP3 oligomerization for establishing persistent inflammatory states. Their effect in differentiating macrophages to attain suppressive phenotypes regulates the cellular tumorigenic potential. Within M2 macrophages, the induction of IL-6/17 cytokines triggers signaling events that activate key transcription factors like AP1, CEBP-β/δ, Erk1/2, and NFkB. These signaling events are pivotal in upregulating the expression of genes like cyclin-D1, VEGF, c-myc, and Bcl-xL that fosters immune regulatory mechanisms and supports tumor expansion. Among the activated transcription factors, the upregulation of the master regulator, NFkB, is critical to drive the assembly of inflammasomes (68). The increased levels of NLRP3, ASC, and caspase-1 assemble to cleave the primitive forms of inflammatory mediators and generate mature forms of IL-1β and IL-18. Subsequently, these inflammatory mediators are released through the pores formed by GSDMD protein, which creates a pro-inflammatory environment in inflamed cells, resulting in the lytic cell death (69). Being a central player, macrophage heterogeneity during inflammation is shaped by the duration, intensity, and type of inflammatory stimuli. Consistent stimuli generate both pro- and anti-inflammatory effects. In alveolar macrophages, hyper-inflammatory responses activate NLRP3, which mediates the stabilization of the immunosuppressive phenotype (70). Since the biological activity of IL-6 relies on STAT-3 activation, studies in BMDMs have demonstrated the physical interaction and the colocalization of STAT3 and NLRP3 following stimulation with nigericin, indicating the IL-6-mediated NLRP3 activation in macrophages (71). Consistent with this finding, IL-6R^-/-^ mice significantly showed dampened chronic inflammation in sterile inflammation mice models, sufficing roles of IL-6 in NLRP3 activation (72). Similar to IL-6, NLRP3^-/-^ and Caspase-1^-/-^ lead to the significant reduction in the pathogenic Th17 responses and IL-17 levels, thereby attenuating inflammation. On the contrary, mice models with hyperactivated NLRP3 mutations showed a Th17-dominant phenotype and inflammasome hyperactivation, suggesting IL-17’s role in NLRP3 activation and assembly (73). In lung cancer, studies have shown upregulation of inflammasome components like IL-1β and IL-18 in the serum of lung cancer patients as compared with adjacent lung tissues. According to the authors, NLRP3 and AIM2 inflammasomes were highly detectable in LUAD patients, whereas other inflammasomes were undetectable. On the contrary, LUSC and SCLC patients had low and moderate expression of NLRP3, respectively. Expression-based analysis of inflammasome-related genes has indicated the differential expression of gene patterns in healthy and lung cancer patients. Moreover, in a mice model, NLRP3 deletion inhibited lung tumorigenesis by reducing the levels of IL-1β, whereas its activation upregulated signaling pathways like PI3K-AKT, Erk1/2, and STAT3, which altogether contributed to increased proliferation and cancer metastasis. Being the downstream effectors in the inflammasome pathway, higher expression of GSDMD correlates with the pathophysiology of lung cancer, resulting in increased tumor size, advanced stages, and reduced survival rates in LUAD and LUSC patients. However, knocking down of GSDMD showed an increase in the levels of metalloproteinases that facilitated apoptosis in lung cancer cells. Utilizing the NLRP3 inhibitor, MCC950, on lung cancer cells, has been shown to delay the tumorigenic process and immobilize anti-tumor responses in cancer patients (74, 75). Altogether, this suggests how the synergistic effect of gut-modulated pro-inflammatory cytokines, IL-6/17, in upregulating NLRP3 and associated signaling mechanisms, all of which culminate to progress tumor development at later stages (**Figure 3**).

## 4 Gut microbiome as therapeutics in lung cancer

Since the role of symbiotic microbiota is linked to modulating the tumorigenic potential of transformed cells; it has been widely explored as a potential diagnostic biomarker to better understand lung cancer and predict patient outcomes (76, 77). It has also been widely adopted to understand lung cancer development and predict clinical prognosis. The antibiotic resistance as a result of conventional therapies has led to chemotherapeutic resistance, posing serious illnesses to human population. To rescue these effects, microorganisms have been engineered and harnessed for their therapeutic potential to overcome drug non-specificity. Recent investigations have identified gut microbiota as a potential source of biological signatures capable of modulating the efficacy of cancer immunotherapies. In a clinical study focused on patients with advanced non-small cell lung cancer (NSCLC) undergoing navumab treatment, a correlation was observed between patient response and gut microbiome characteristics. Specifically, responders exhibited increased intestinal microbial diversity, which was associated with improved progression-free survival rates. Additionally, the study by Peng Song et al., showed the ability of *Methanobrevibacter* and *Parabacteroides* in ameliorating the antitumor activities to PD-1 inhibitors. Similarly, the administration of *Bifidobacterium* in mice has shown to enhance the functionality of dendritic cell and the migration of cytotoxic T-cells to the tumor site, exhibiting antitumor activities and anti-PD-1 efficacy (78, 79). Being an intestinal representative, *Bifidobacterium* exerts protective effects against LPS and TNF-α induced inflammatory responses, conferring anticancer effects to the growing tumor mass (80). These findings indicate that alterations in gut microbiota diversity represent a potentially critical determinant of therapeutic responses that prolongs NSCLC progression-free survival rates in patients undergoing treatment. While, how the potency of these gut microbiota can be leveraged to resist tumor growth in lungs needs to be addressed?

When it comes to exploiting the power of gut microorganisms for achieving therapeutic responses in lung cancer, several strategies have been employed, all of which ultimately aims towards microbial modulation (81). Among the most commonly used approaches including additive therapy, subtractive therapy, and modulatory therapy, modulating microbiotal physiology and stimulating their inherent therapeutic traits simulate biological mechanisms towards immune responsive states, and therefore the modulatory therapy works best to achieve therapeutic effects. In additive therapy, microorganisms bearing potential therapeutic traits are added to overcome the effect of deleterious pathogens and restore gut homeostasis. In contrast, subtractive therapy involves eliminating the potential threat causing microorganisms to balance healthy microbes in the gut environment (82). As discussed, the lung surfaces in cancer are dominated by opportunistic pathogens that reduce the levels of symbionts like *Escherichia, Enterobacter*, *Dialister, Kluyvera, Faecalibacterium*, leading to dysbiotic events in the gut. It has been evidenced that not the whole microorganism, rather a pathogenic component of these microbiota is sufficient to induce immune mechanisms, favoring the sustenance of immune modulatory phenotypes towards inflammatory responses in the lung epithelium. This initiates signaling events that culminate to stimulate the LPS-induced MyD88 dependent production of IL-1β and IL-23 that differentiates IL-17-producing innate lymphoid cells (83) to produce IL-6 and IL-17 during tumor-promoting events. What if we engineer these microbes to reverse the pathogenic effect of inflammatory cytokines for immune responsive mechanism and modulate the inflamed tumor site through enriching microbial populations in counteracting immune suppressive events in lung epithelial cells?

### 4.1 Synthetic biology: recent examples, technical feasibility, and safety concerns

**Figure 4:**
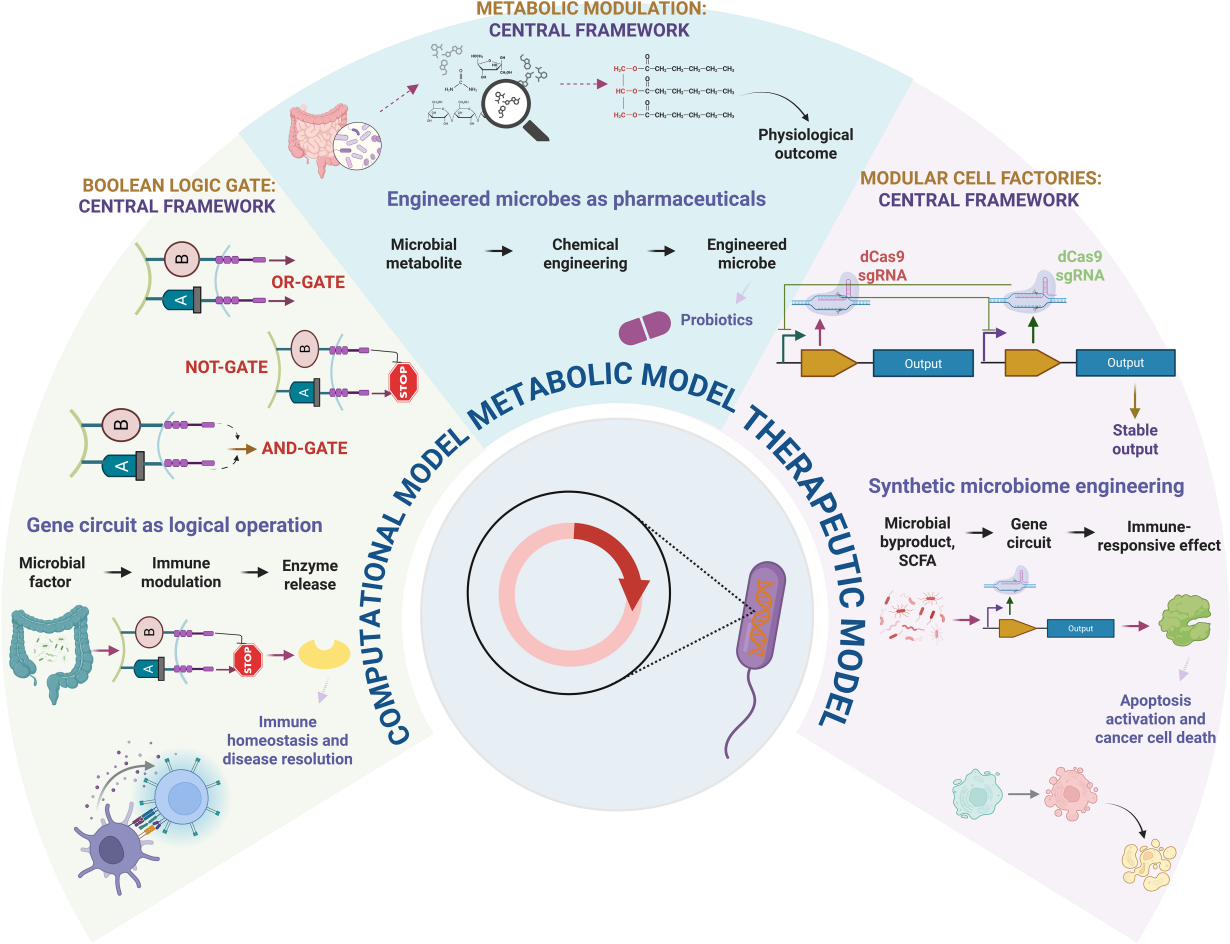
Proposed model of synthetic biology-based interventions. The figure illustrates different models used for microbial engineering, involving logic gates, synthetic circuits, and metabolic engineering principles. All these strategies aim to discover immune-responsive effect, helping to resolve cancer-associated immune regulation.

Microbial consortia are naturally occurring communities of diverse microorganisms, which are prevalent and hold significant value across various bioeconomical sectors. Compared to cultures with just one type of microorganism, these groups are better at handling environmental challenges, have a wider range of metabolic abilities, and interact with each other in ways that help them communicate. These intrinsic features make microorganisms an excellent platform for synthetic biologists, who aim to engineer them for scientific applications (**Figure 4**). One of the biggest advantages of leveraging bacterial systems for delivery is their ability to locally target tissues, which lessens systemic therapeutic exposure. Over other entities, bacteria have been chosen to deliver drugs to their resident sites mostly, yet it is still difficult to therapeutically target oral routes and tumor centers (84). The field of synthetic biology is gaining more attention because many organisms can be easily modified with large DNA, allowing scientists to add complex biological processes to study how they affect signaling pathways (85). Leveraging synthetic biology for bioengineering gut lifeforms opens promising avenues for developing novel probiotics that physiologically enhance lung health. One of the primary applications by which engineered microorganisms offer health benefits is through synthetically constructing biosynthetic pathways that aim to increase metabolite production by the gut bacteria. Synthetic development of microbiota-based interventions covers everything from recombinant probiotics to advanced engineering principles that, being host-responsive, can detect and sense varied environmental cues (86, 87). This could be achieved through adopting gene editing tools like CRISPR-Cas9 that precisely modulate bacterial genomes and allow for targeted editing to rescue adverse effects (88). Presently, the CRISPR-Cas-based system adopts a subtractive approach to eliminate harmful pathobionts, which holds promise towards new antimicrobial drug design. Aforementioned that *Enterobacter*, *Escherichia*, and *Veillonella* define the diseased lung phenotype; engineering these entities helps enhance their therapeutic traits by mitigating their pathogenic abilities. As an effect, the transcriptional control in the production of pathogenic enzymes optimizes gut function that facilitates the destruction of harmful toxins in restoring the microbial pool (89). The synthetic bioengineering of these strains tailors individual needs by improvising the probiotics potential of gut pathobionts, addressing dysbiotic events and pathological states in lungs. For instance, these commensals equilibrate the levels of required inflammatory cytokines, offering potential treatment for cancer. Moreover, they can also produce antimicrobial peptides or break down harmful metabolites (90), offering a selective approach to target pathogenic bacteria while preserving beneficial species, thereby supporting the maintenance and restoration of a balanced microbial ecosystem. Additionally, the approach entails creating gut monitoring systems by integrating biosensors into microbes, where the engineered microbes respond to fluctuations in their environment, thereby providing information indicative of gut dysbiosis. This targeted strategy improves the efficacy of treatments and minimizes potential side effects by ensuring that the therapeutic interventions are specifically designed for each patient’s unique microbiome (91). Consequently, this personalized method represents a significant advancement in integrative health care, particularly for conditions affecting both the gut and lungs.

**Figure 5:**
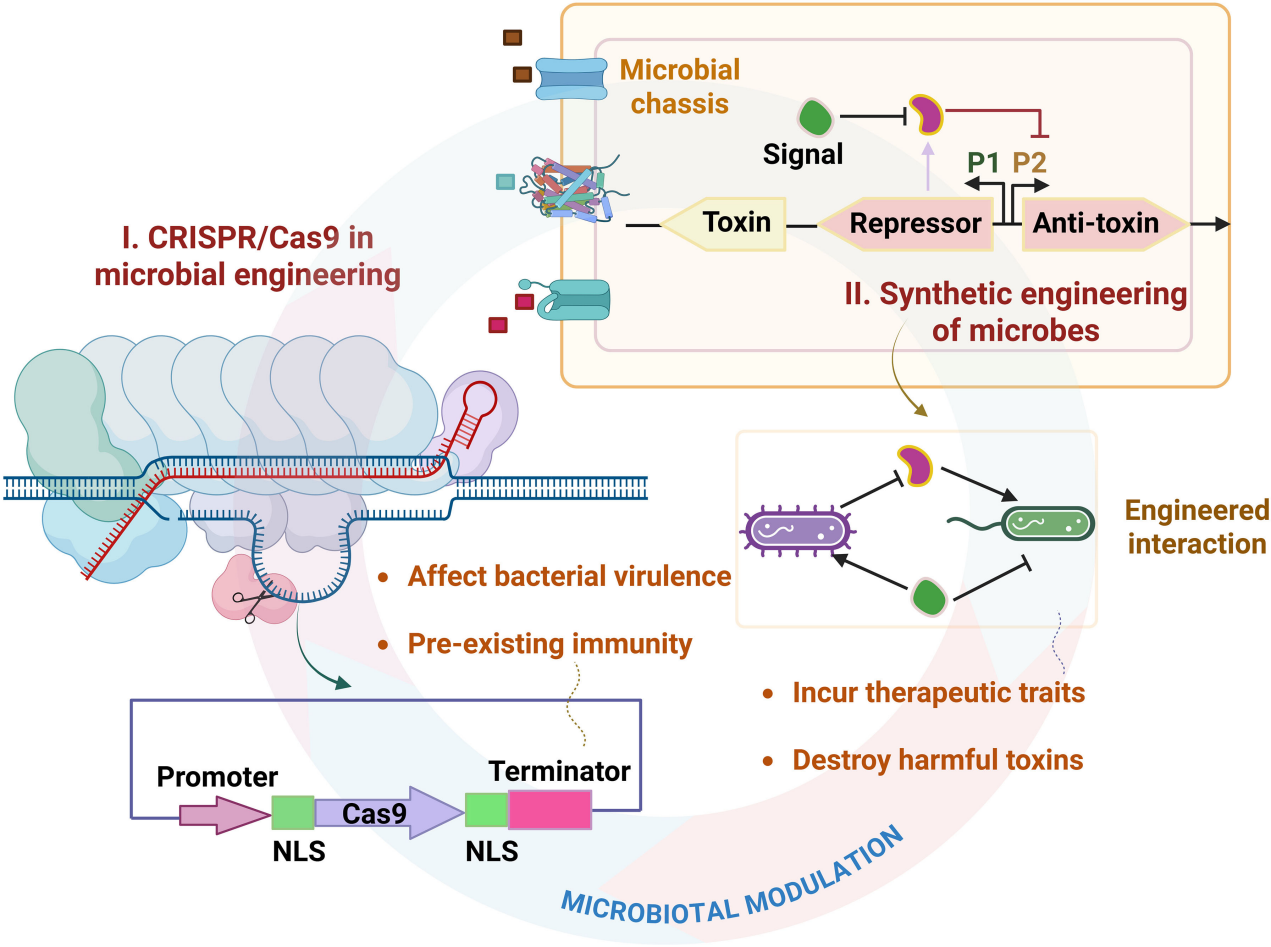
Synthetic microbial engineering-transcriptional control mechanisms to modulate microbial materials. Microbial modulation is achieved through two approaches: Utilizing CRISPR/Cas9 systems allows editing bacterial genomes to treat disease pathologies, such as cancer. In a bottom-up approach, isolates are engineered for enhanced anti-toxin production, offering therapeutics against harmful invaders. Both approaches aim towards engineered microbe interaction, rescuing deleterious effects of their metabolites.

The potential of synthetic biology to meet global needs is enormous. Researchers have created novel therapies using microbial chassis built from existing biological pathways with newer constructs for gene sensing and the production of medical biomolecules. Synthetic development of Januvia, which is a diabetic drug from Merck, was designed to increase the insulin production by inhibiting the enzyme dipeptidyl peptidase 4. Since its discovery, it has been prescribed to diabetic patients for resolving disease. Similarly, HEK-293-β cells were artificially constructed for glucose-response insulin synthesis, which, when implanted peritoneally into mice, maintained glucose homeostasis and corrected diabetic hyperglycemia in 3 days of delivery. PROVEN, a biological nitrogen fertilizer developed by Pivot Bio, is the first biological fertilizer based on proteobacterium that fixes nitrogen in corn roots. Herein, synthetic biology was exploited for genome remodeling that switched on the genes responsible for nitrogen fixation. CARs are engineered receptors that contain both T cell-activating and antigen-binding domains. T-cells isolated from patients are ex vivo engineered to express a specific CAR, followed by transferring back to the patient who eliminates cancer antigens. Kymriah, a treatment for B-cell acute lymphoblastic leukemia by Novartis is the first CAR-T therapy to acquire FDA approval, which was designed to express an antibody to specifically target CD19 antigens on cancer cells. The results are promising, and almost ~700 CAR-T therapies are in trial, mostly targeting hematological cancers, solid tumors, viral infections, and autoimmune diseases (92, 93). During the COVID-19 pandemic, the development of an RNA-based vaccine by Novartis is one of the newsworthy examples of synthetic biology. Artificial receptors created using synthetic engineering are used in medical applications for controlling transducing signals, providing the possibility of controlled signaling in living cells. Examples include the development of synNotch, wherein the transmembrane and the intracellular domain remain intact; however, the engineered extracellular domain senses nanobodies, capable of recognizing agents to transduce signaling events. Synthetic biology is also leveraged to engineer iPSCs for medical applications. The use of ex vivo cardiomyocytes to generate fetal hearts via Yamanaka’s factors is one such example. Most importantly, the field aims to repair damaged tissues during disease pathophysiology. In cancer, the overexpression of Bcl-2 leads to tumorigenic risks. CRISPR/dCas9 bioswitches help to resolve the problem via spatial gene expression. Alternatively, the Tet repressor-based system could work well by adopting the Tet-off system for controlling exaggerated tumor responses (94). While the field of synthetic biology holds promise, there are still the associated challenges. Since it is leveraged to engineer living organisms, the field encounters technical dichotomy, wherein the technology can be exploited for good purpose or can be misused. Although it is difficult to eliminate the biological abuse of synthetic biology, risks can be minimized by well-formulating biosafety and biosecurity measures. Biosafety concerns may include the intrinsic microbial ability to cause disease of any severity in living organisms. Usually, a single gene manipulation is easier than manipulating multiple genes in a pathway, which naturally is complex and poses risks to human health. The release of synthetically engineered organisms in an environment may disrupt ecological imbalance since it substitute native microflora. However, being synthetic, they are preyed upon by natural inhabitants. Additionally, with the advent of molecular engineering, the possibility of bioterrorism is expanding. Owing to genetic sequence availability in databases, the chemical synthesis has achieved high success rates. Several studies indicate the off-target effects resulted as an effect of CRISPR/Cas9 genome editing, which could lead to potential health consequences. However, Hajian et al. developed the CRISPR-chip platform that utilized immobilized dCas9 coupled with sgRNA for fast detection of target nucleic acids (**Figure 5**). The field also requires knowledge and an understanding of bioethical principles. As per the principle, the development of synthetic biology should be ensured in a fair, ethical way, which includes justice, responsibility, public betterment, and democratic deliberation (95). Ensuring stable conformations of engineered microbes in humans, preventing their cross-talks with native organisms, and mitigating regulatory effects needs proper attention to achieve goals of microbial engineering (96). Since the field employs providing high therapeutic potential, the genetic control and the stability of engineered microorganisms are of great importance, which needs to be addressed to further accelerate the power of microbiome-based therapeutics (97).

## Discussion

**Figure 6:**
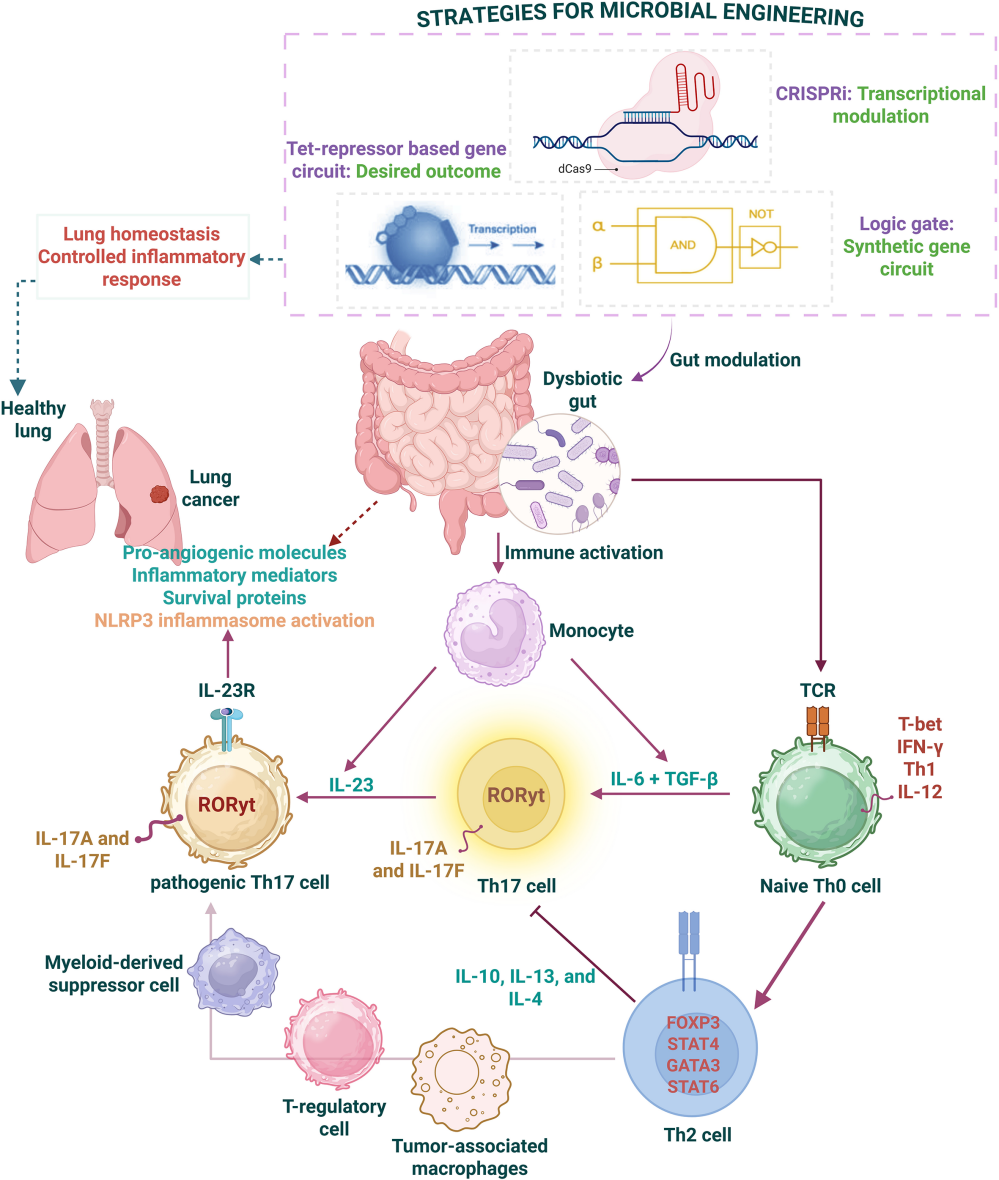
Figure summarizing the role of gut dysbiosis, inflammatory events, and microbial engineering. Immune activation in gut dysbiosis activates immune effectors, releasing cytokines, which culminate to modulate lung homeostasis. The combination effect of IL-6/17, immunosuppressive cytokines and immune cells releases pro-angiogenic molecules that contribute to processes associated with lung cancer. However, adopting different strategies for microbial engineering can help achieve a controlled inflammatory response, resolving disease outcomes.

Because lung cancer accounts for the highest deaths worldwide, it has raised a significant concern around the health of both sexes. Numerous factors, including smoking, occupational exposure, and many more, contribute to rising cancer incidences, of which the imbalances in gut microbiota have led to lung cancer development. Being similar in embryological origin, dysbiotic events in the gut reshape lung developmental programs. A few of the varied microflora inhabiting the gut also home to lung sites. The similarity in the microbiotal communities facilitates the homing of gut commensals to the lungs. Environmental stressors, be they pathogen, stress, pH, or other factors, induce gut pathobionts by inducing signaling events that propagate inflammatory responses by homing to other sites. Among the known biological interactions of the gut with other organs, the gut-lung axis is understood to favor lung neoplastic events. Homeostatic imbalances in the cytokines contribute to inflammatory processes, of which IL-6 and IL-17 exemplify oncogenic events. Consequently, the elevated levels of circulating tumor cells exemplify oncogenic responses, which are associated with poor survival and therefore serve as prognostic markers, as seen in breast cancer patients (98). IL-6 belongs to the broad class of cytokines that act intrinsically on cells through distinct signaling pathways to sustain tumor growth. A lesser role for IL-6 has been evidenced in which it displays an anti-tumor effect. Accumulating evidence establishes the role of IL-6 in invasion and metastasis during activated immune responses. Majorly, IL-6 mobilizes anti-tumor immune response by supporting the activation, expansion, survival, and proliferation of T-cells, increasing CD8+ T-cell trafficking to the tumor site. In immunization settings, IL-6 is required for T-cell priming, induction of protective IFN-γ response, regulating immunosuppressive phenotypes like T-reg, and providing help to B-cells. Nonetheless, IL-6 prevents apoptotic death of T-cells by increasing the levels of Bcl-2 and Bcl-xL. A study defines the immunostimulatory role of IL-6 in skewing CD4+ T-cells towards the Th17 phenotype. In a mice model with aggressive melanoma, IL-6 has been shown to alleviate T-reg-mediated immune responses for effective priming of cytotoxic CD8+ T-cells. Under thermal stress, the IL-6 level elevates, and its trans-signaling mechanism enhances lymphocyte homing to tumor sites in cancer patients and mice, signifying the bioavailability of IL-6 during acute inflammation or initial phases of infection. On the contrary, IL-6 exhibits pro-tumor properties by influencing the survival, proliferation, and metastatic potential of tumor cells. Higher levels of IL-6 correlate with immunosuppression while elevating the counts of immunomodulatory phenotypes. Studies reveal elevated levels of IL-6 as a prognostic indicator associated with poor outcomes in multiple tumor types, including lung cancer. Disruption of IL-6 signaling delays tumor development in a murine inflammation model, suggesting the role of IL-6 not only in initiation, but also during neoplastic transformation. In the TME, the molecules secreted by tumor cells stabilize Th2 subsets that release IL-6, which act in an autocrine feedback loop to support tumor development. Acting through STAT3, IL-6 sustains tumors by elevating the levels of anti-apoptotic proteins like Bcl-2, Bcl-xL, and survivin. In breast cancer, direct STAT3 signaling is shown to increase the levels of survivin, which is important to maintain hypoxic states in the TME (99–101). Like IL-6, the paradoxical role of IL-17 regulates malignant transformation. As mentioned before, IL-6 skews the CD4+ T-cell population to elevate IL-17 levels; IL-17 A/F, released by the Th17 subset initially, exhibits anti-tumorigenic roles. Interestingly, in immunocompetent mice models, the ectopic expression of IL-17 in tumor cells enhanced anti-tumor immune responses. However, the reverse is true for immunodeficient mice, where IL-17 promoted metastatic spread via releasing angiogenic mediators. Owing to decreased NK cell population and lesser T-cell production of IFN-γ, it exerts an immunosuppressive effect. Studies demonstrated that of all T-cell populations, γδ Th17 cells are instrumental in mediating tumor growth in many cancer subtypes. In breast cancer, they drive the expansion of tumor-associated neutrophils, impeding the roles of cytotoxic T-cells. Furthermore, their role has been implicated in recruiting alternative macrophages expressing higher levels of IL-17R, promoting the proliferation of ovarian cancer cells. This suggests Th-17 secreted IL-17-mediated protective and pathological roles during tumor development (102–104). The pleiotropic feature of these cytokines activates the inflammasome that inflames the infected site towards tumor progression (**Figure 6**). What if we reduce the levels of these cytokines to combat cytokine synergism? This requires a profound understanding of genetic algorithms that allows microbial bioengineering to achieve the goal. To date, synthetic biology stands out as an innovative approach to refurnishing healthcare systems with transformative goals that harness bioengineering principles towards microbial chassis. Synthetic microbiota can be leveraged for healthcare systems, starting from delivering therapeutic molecules like cytokines, bioactive peptides, bacteriocins, and enzymes into the lungs that help achieve pulmonary homeostasis by modulating biological systems (105, 106). Unfortunately, the field requires addressing ethical rules, regulatory mechanisms, and technical fidelity. The established groundwork and the ongoing research in the microbiotal signaling axis have witnessed the translational power of microbiome-based therapeutics that will evolve to tailor individualized needs for inflammatory diseases in the future.

## Glossary

AKT: AK strain Transforming
AP1: Adaptor Protein 1
ASC: Apoptosis-associated Speck-like protein containing a Caspase recruitment domain
CAR: Chimeric Antigen Receptor
Cas9: CRISPR-associated protein 9
CEBP-β/δ: CCAAT/Enhancer-Binding Protein- Beta and Delta
CRISPR: Clustered Regularly Interspaced Short Palindromic Repeats
DAMPs: Damage-Associated Molecular Patterns
DC: Dendritic Cell
EMT: Epithelial-to-Mesenchymal Transition
Erk1/2: Extracellular signal-regulated kinase 1/2
FOXP3: Forkhead bOX P3
GSDMD: GaSDerMin D
GATA-3: GATA binding protein-3
GI: GastroIntestinal tract
IBD: Inflammatory Bowel Disease
ICIs: Immune Checkpoint Inhibitors
IELs: IntraEpithelial Lymphocytes
IFN-γ: InterFeroN-γ
IL: InterLeukin
ILCs: Innate Lymphoid Cells
iPSC: induced Pleuripotent Stem Cell
JAK: JAnus Kinase
LPS: LipoPolySaccharide
LUAD: LUng ADenocarcinoma
LUSC: LUng Squamous Cell carcinoma
MAPK: Mitogen-Activated Protein Kinase
MDSC: Myeloid Derived Suppressor Cell
NFkB: Nuclear Factor kappa-light-chain-enhancer of activated B-cells
NK: Natural Killer cell
NKT: Natural Killer T cell
NLRP3: NOD-Like Receptor Pyrin domain containing 3
NSCLC: Non-Small Cell Lung Cancer
PAMPs: Pathogen-Associated Molecular Patterns
PD-1: Programmed cell Death protein-1
PI3K: PhosphoInositide 3-Kinase
PRR: Pathogen Recognition Receptor
RORγt: RAR-related Orphan nuclear Receptor gamma t
SCFAs: Short Chain Fatty Acids
SCLC: Small Cell Lung Cancer
SOX2: SRY-bOX transcription factor 2
SRC: Proto-oncogene tyrosine-protein kinase
STAT: Signal Transducer and Activator of Transcription
TAM: Tumor-Associated Macrophage
TGF-α: Tumor Growth Factor-alpha
Th: T-helper
TME: Tumor MicroEnvironment
TNF-α: Tumor Necrosis Factor-alpha
T-reg: T-regulatory cell
VEGF: Vascular Endothelial Growth Factor
YAP: Yes-Associated Protein
